# Induction of T helper 1 response by immunization of BALB/c mice with the gene encoding the second subunit of *Echinococcus granulosus* antigen B (EgAgB8/2)

**DOI:** 10.1051/parasite/2012192183

**Published:** 2012-05-15

**Authors:** H. Boutennoune, A. Qaqish, M. Al-Aghbar, S. Abdel-Hafez, K. Al-Qaoud

**Affiliations:** 1 Department of Biological Sciences, Yarmouk University Irbid Jordan

**Keywords:** *Echinococcus granulosus*, EgAgB8/2, gene immunization, IgG1/IgG2a ratio, T helper 1, *Echinococcus granulosus*, EgAgB8/2, vaccination génique, rapport IgG1/IgG2a, T helper 1

## Abstract

A pre-designed plasmid containing the gene encoding the second subunit of *Echinococcus granulosus* AgB8 (EgAgB8/2) was used to study the effect of the immunization route on the immune response in BALB/c mice. Mice were immunized with pDRIVEEgAgB8/ 2 or pDRIVE empty cassette using the intramuscular (i.m.), intranasal (i.n.) or the epidermal gene gun (g.g.) routes. Analysis of the antibody response and cytokine data revealed that gene immunization by the i.m. route induced a marked bias towards a T helper type 1 (Th1) immune response as characterized by high IFN-γ gene expression and a low IgG1/IgG2a reactivity index (R.I.) ratio of 0.04. The i.n. route showed a moderate IFN-γ expression but a higher IgG1/IgG2a R.I. ratio of 0.25 indicating a moderate Th1 response. In contrast, epidermal g.g. immunization induced a Th2 response characterized by high IL-4 expression and the highest IgG1/IgG2a R.I. ratio of 0.58. In conclusion, this study showed the advantage of genetic immunization using the i.m. route and i.n. over the epidermal g.g. routes in the induction of Th1 immunity in response to *E. granulosus* AgB gene immunization.

## Abstract (fr)

Un plasmide (pDRIVE-EgAgB8/2) contenant le gène codant pour la seconde sous-unité de l’antigène B d’*Echinococcus granulosus* (EgAgB8/2) a été utilisé pour étudier l’effet de la voie d’immunisation sur les réponses humorale et à médiation cellulaire chez des souris BALB/c. Les souris ont été immunisées avec le plasmide recombinant pDRIVE-EgAgB8/2 ou avec le plasmide vide pDRIVE en utilisant les voies d’immunisation suivantes : intramusculaire (i.m.), intranasale (i.n.) ou épidermale via un pistolet à gènes (g.g.). L’analyse de la production des anticorps et des cytokines a révélé que l’immunisation par ADN par la voie i.m. a induit une réponse immunitaire de type T helper 1 (Th1) qui se caractérise par une haute expression du gènes IFN-*γ* et un faible rapport d’IgG1/IgG2a (0,04). La voie i.n. a montré une expression modérée d’IFN-*γ*, mais un rapport plus élevé d’IgG1/IgG2a (0,25), indiquant une réponse Th1 modérée. En revanche, l’immunisation épidermale par le g.g. a induit une réponse Th2 caractérisée par la haute expression l’IL-4 et le rapport le plus élevé d’IgG1/ IgG2a (0,58). En conclusion, cette étude a montré l’avantage de l’immunisation par ADN en utilisant les voies i.m. et i.n. par rapport à la voie g.g. dans l’induction de l’immunité Th1 en réponse à l’immunisation par l’ADN codant l’antigène B d’*E. granulosus* (AgB).

Cystic echinococcosis (CE) or unilocular hydatidosis is a major cosmopolitan zoonotic disease caused by the infection with the larval stage (hydatid cyst) of the tiny dog tapeworm, *Echinococcus granulosus* which cycles between a carnivorous definitive host (mainly dog) and herbivorous livestock intermediate hosts. Humans appear in the cycle as accidental host after ingesting food contaminated with parasite eggs (Eckert & Deplazes, 2004). CE affects the liver and the lungs mainly but other organs such as the spleen, pancreas, abdominal and pelvic cavities, musculoskeletal system, kidneys, heart and even the brain can be affected. The infection leads to morbidity and mortality worldwide and represents a significant hazard in most developing countries, where it impairs human health and considerable loss in animal production (Eckert & Deplazes, 2004).

Studying the immunological profile of human patients with different stages of hydatid disease revealed that parasite survival is always supported with Th2 lymphocyte polarization of host’s immune system (Riganò *et al.*, 2004). Conversely, induction of a Th1 response may lead to protection against successful parasite establishment. This can be achieved by vaccination with appropriate parasite antigens using specific immunization routes (Al-Qaoud *et al.*, 2008; Ding *et al.*, 2008). Recently, DNA vaccination to viruses, bacteria and parasites has been used as a tool to study the effects of the immunization route (parenteral, intranasal, oral, intralymphatic and intrahepatic) on the immune response (Fynan *et al.*, 1993; Chen, 1998; Sasaki *et al.*, 1998; Maloy *et al.*, 2001; Cruz-Revilla *et al.*, 2006). This approach involves the direct introduction of plasmid DNA carrying the gene encoding the antigenic protein of interest, which is then expressed within cells of the immunized animal.

Application of this new vaccination technology with regard to parasitic infection provides new hope for significant advances in anti-parasitic vaccine research. The multiplicity of parasite stages that the host is exposed to and the complexity of each stage characterizes most parasitic infections as being chronic and associated with immunosuppression (Ivory & Chadee, 2004).

Gene immunization against *E. granulosus* infection has been attempted to modulate the immune system toward a Th1 protective response by the use of cytokine genes along with Eg95 (an oncospheral protein product) (Scheerlinck *et al.*, 2001). Another target antigen is antigen B (AgB), which is a polymeric lipoprotein and major component of hydatid cyst fluid. It is a strong immunogenic protein, which is believed to be involved in the modulation of the host immune response (Siracusano *et al.*, 2008). At the molecular level, AgB is encoded by a gene family of at least five genes each of which codes for 8 kDa subunit (Mamuti *et al.*, 2006; Muzulin *et al.*, 2008). As the second subunit of antigen B (EgAgB8/2) showed the highest sensitivity and specificity, a murine expression vector containing the open reading frame of this subunit was designed and used for immunization in combination with cytokine genes (Al-Aghbar *et al.*, 2008). This study was designed to investigate the effect of gene immunization route using pre-designed plasmid containing EgAgB8/2 on the immune response of BALB/c mice. The relevance of this approach to *E. granulosus* infection is based on the fact that the egg infective stage of the parasite enters the intermediate host and humans through the oral or nasal ports of entry rendering the mucosal lining as the first line of contact with the parasite.

## Materials and Methods

### Preparation of crude sheep hydatid fluid

Crude sheep hydatid fluid (CSHF) was aspirated from fertile hydatid cysts obtained from *E. granulosus* infected livers and lungs of sheep slaughtered at local abattoir in Irbid, Jordan. CSHF was centrifuged at 1,000 × g for 15 min and the supernatant was dialyzed against phosphate buffered saline (PBS, pH 7.2) then filtered using a millipore filter (0.2 μm), lyophilized using Edward, EF Modulyo lyophilizer (Crawley, United Kingdom) and stored at -20 °C until use. Following reconstitution of the lyophilized material at 2 mg/mL with phosphate buffer saline (PBS) (pH 7.2), the protein concentration was determined using Bradford method (Bradford, 1976).

### Preparation of AgB

Native AgB was prepared from a pool of CSHF based on the method of Oriol *et al.* (1971) with some modifications. Briefly, 50 mL of CSHF were dialyzed against 0.005 M acetate buffer (pH 5) overnight at 4 °C and centrifuged at 13,000 × g for 30 min (Sigma, USA). The precipitate was dissolved in 5 mL of 0.2 M phosphate buffer (pH 8), autoclaved for 15 min at 15 psi and 120 °C and centrifuged at 13,000 × g for 60 min. The supernatant containing AgB was filtered by a millipore filter (0.2 μm), aliquoted and stored at -20 °C until use.

### Plasmid DNA preparation

A recombinant plasmid pDRIVE-EgAgB8/2 was constructed as previously described (Al-Aghbar *et al.*, 2008). The empty cassette plasmid (pDRIVE) and recombinant plasmid (pDRIVE-EgAgB8/2) were propagated in *E. coli* DH5α and purified using Wizard^®^ Plus Maxipreps DNA Purification kit (Promega, USA) to remove possible endotoxin contamination. The concentration of each plasmid DNA was determined spectrophotometrically at 260 nm and stored at -20 °C until used.

### Preparation of DNA gold microcarriers

DNA-coated gold beads for Gene Gun immunization were prepared according to the manufacturer instructions (BIO-RAD, Hercules, CA). 2 μg/μL of each purified expression vector or the empty cassette plasmid were precipitated on 1.6 μm gold particles (BIO-RAD, Hercules, CA) by 0.1 M spermidine (Sigma, Saint Louis, MO) followed by 2.5 M CaCl_2_. DNA-gold particles were resuspended in 0.05 mg Polyvinylpyrrlidone (PVP)/ethanol then precipitated inside Tefzel gold-coat tube (BIO-RAD, Hercules, CA) and dried using nitrogen gas.

### DNA immunization

48 six-eight weeks old female BALB/c mice bred at Yarmouk University animal house facilities were divided into six groups (eight mice/group). Three groups were primed with pDRIVE-EgAgB8/2 plasmid DNA either intramuscularly (200 μg/mouse), intranasally (200 μg/mouse) or epidermally using the Helios Gene Gun (2 μg/mouse) route. Animals were boosted four times, and sacrificed 15 days after the last injection (50 days after the first immunization). The first booster was given 14 days following the initial immunization while the three other boosters were given at one week interval from each other. Intramuscular injections were given in the quadriceps muscle of the thigh of each mouse while for intranasal immunization mice received 31 μL of plasmid DNA (200 μg) to the nostril. Gene gun immunization was done by shooting the bullet contents of plasmid DNA into a shaved skin of mouse abdomen. Three comparable control groups were immunized with empty cassette plasmid (pDRIVE) using the relevant route. Blood was collected at day of experiment termination (50 days after the first immunization), and the sera were prepared and stored at -20 °C until use.

### Analysis of AgB-specific antibodies by ELISA

For serum antibody (Ab) screening, standard Enzyme Linked Immunosorbent Assay (ELISA) protocol was performed. Briefly, flat bottomed 96 well polystyrene microtiter plates (Greiner, Germany) were coated with 100 μL of 10 μg/mL AgB in carbonate-bicarbonate buffer, pH 9.6 overnight at 4 °C, then blocked with 100 μL of 2% bovine serum albumin (BSA) in PBS for one hour at room temperature (RT). 100 μL of serum samples from each mouse diluted at 1:100 in 1% BSA were added in duplicates and incubated one hour at room temperature (RT). Positive control (pooled hyperimmune serum prepared from AgB immunized mice) and negative control sera (serum from PBS immunized mice) were incorporated in each plate. After that, horse radish peroxidase (HRP) conjugated to goat antimouse IgG subclasses (IgG1, IgG2a, IgG2b or IgG3) (Sigma, St Louis, USA) diluted at 1:500 in 1% BSA were added to each well, and plates were incubated for one hour at RT. Following each step, wells were washed with 0.05% Tween 20 (Sigma, USA) in PBS. Finally, 100 μL of 0.1% O-phenylenediamine (Sigma, USA) containing hydrogen peroxide in 0.1 M citrate buffer (pH 4.5) were added to each well and the (O.D.) was measured at 490 nm using DigiScan ELISA Reader. Antibody titers were expressed as mean reactivity index (R.I.) as described earlier (Al-Aghbar *et al.*, 2008).

### Quantification of IFN-γ and IL-4 mRNA by real-time PCR

The cytokine gene expression profile of immunized mice was evaluated by real time-PCR. RNA was isolated from splenocytes using TRIzolTM reagent (Sigma, USA) and first-strand cDNAs were synthesized using Super ScriptTM III Reverse Transcriptase. RoterGene 3000A Cycler (Corbett Research, Australia) was used to amplify and analyze the expression of cytokine genes relative to β-actin gene as internal control.

For the purpose of cDNA amplification, SYBER-Green I dye was used. For PCR analysis, the following primer pairs (Liu *et al.*, 2005) were used: for IFN-γ, forward primer 5’ CCT GCA GAG CCA GAT TAT CTC T 3’ and reverse primer 5’ TCG CCT TGC TGT TGC TGA AGA A 3’; for IL-4, forward primer 5’ CAC TTG AGA GAG ATC ATC GGC 3’ and reverse primer 5’ TGC GAA GCA CCT GGA AGC CC 3’, and for β-actin, forward primer 5’ CCC CGG GCT GTA TTC CCC TCC A 3’ and reverse primer 5’ TCC CAG TTG GTA ACA ATG CCA 3’ (Alpha DNA, Montreal, Canada).

The PCR reaction mixture contained 6.25 μL of 2 × PCR master mix, 0.5 μL forward and reverse primers (5 μM) for each cytokine, 0.5 μL SYBER-Green (0.125 ×) and 0.5 μL cDNA or plasmid expression vector as positive control or nuclease free water as negative control (Promega, Madison, WI). Nuclease free water was added to complete the volume to 12.5 μL.

The optimization of Real-Time PCR reaction was performed according to the manufacturer instructions as described by Bustin (Bustin, 2000). The amplification steps included initial denaturation at 95 °C for 5 min, followed by 40 cycles of amplification, each includes denaturation at 95 °C for 30 sec, annealing at 56 °C for 30 sec for IFN-γ and IL-4 and 45 sec for β-actin, and extension at 72 °C for 30 sec.

Relative quantification of the cytokine gene expression was calculated using comparative C^T^ method. After determination of the threshold cycle (C^T^), the expression (Ex) and the relative expression level (RExL) of each cytokine was calculated in relation to expression level of β-actin gene as follows:

Ex = 2^∆∆C^T^^
∆∆C^T^ = ∆C^T^ sample - ∆C^T^ normalizer (no template control, NTC)
∆C^T^ sample = C^T^ sample - C^T^ positive control RExL of each cytokine gene = (expression of cytokine gene/expression of β-actin gene) × 100%

### Statistical analysis

Statistical analysis was performed using SPSS version 15.0 software. For each test, the result was expressed as mean ± standard error of mean (M.E.). The Kruskal- Wallis test and one-way analysis of variance (ANOVA) were used to analyze data. A p-value less than 0.05 was considered statistically significant.

## Results

### Intramuscular route achieved highest antigen (Ag) specific igG2a and igG2b

**Fig 1.**
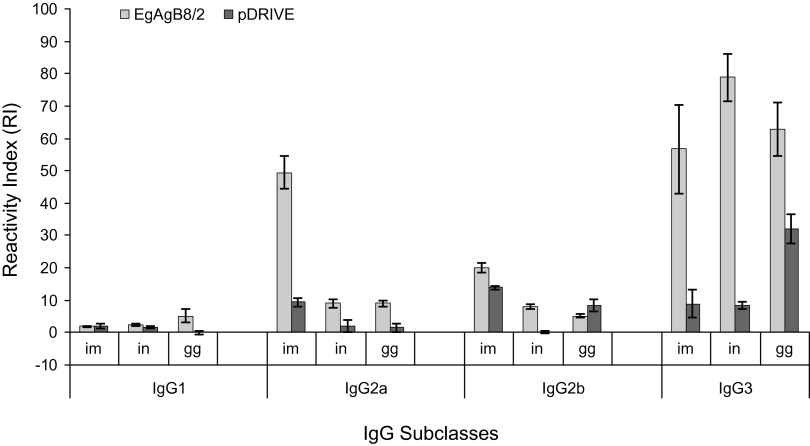
Serum IgG isotype titers (mean ± sd) of mice, obtained 50 days after the first immunization with recombinant EgAgB8/2 plasmid and control or empty plasmid (pDRIVE), using different routes of immunization (i.m., i.n. and g.g.). Specific IgG1, IgG2a, IgG2b and IgG3 titers were determined by ELISA assay against purified AgB. Data are represented in mean value of reactivity index (R.I.) and standard errors of means.

IgG1 presented very low R.I.s in all immunized mouse groups regardless of the immunization route. Mice immunized with EgAgB8/2 gene showed significantly higher IgG2a and IgG2b in i.m. immunization compared to i.n. (p < 0.001) and epidermal g.g. (p < 0.001) routes of immunization. Moreover, a statistically significant difference was observed between i.n. and epidermal g.g. routes of immunization for IgG2b (p < 0.001) but not for IgG2a (p = 0.429). As for the IgG3 response, a significantly higher response was seen in mice immunized intranasally than those immunized intramuscularly (p = 0.008) or through the epidermal g.g. route (p = 0.014) (Fig. 1).

Calculation of IgG1/IgG2a R.I. ratios, using various routes of immunization, are shown in Table I. Evidently, the g.g. route resulted in the highest IgG1/IgG2a R.I. ratio (0.58), while the reverse was seen when the i.m. route was used (IgG1/IgG2a R.I. ratio of 0.04).

**Table I.** AgB-specific IgG1/IgG2a reactivity index (R.I.) ratios in mice immunized with EgAgB8/2-DNA using different routes of inoculations. Serum samples were collected 50 days following initial inoculations and were assayed for AgB-specific IgG1 and IgG2a.

Route of inoculation	R.I. ratio IgG1/IgG2a*
Intramuscular	0.04
Intranasal	0.25
Epidermal gene gun	0.58

* Statistically significant differences in ratios were seen among the three routes.

### Both i.m. and i.n. routes induced high IFN-γ gene expression

**Fig 2.**
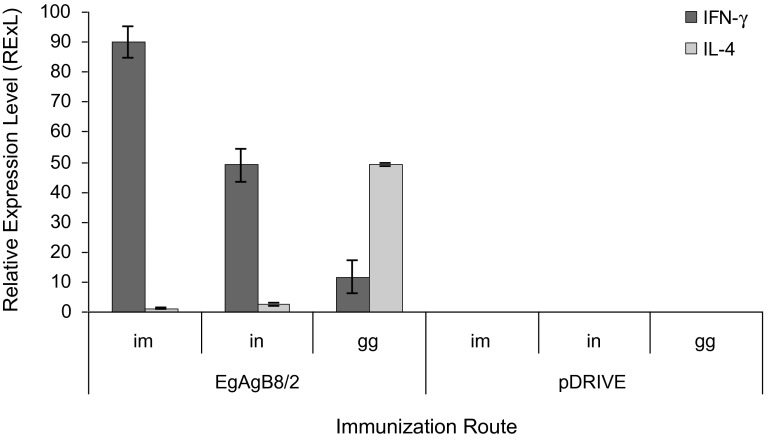
Relative expression levels (RExL.) of IFN-γ and IL-4 genes determined in mice 50 days post-immunization with pDRIVEEgAgB8/ 2 and empty plasmid (pDRIVE), using different routes of immunization (i.m., i.n. and g.g.). mRNA was prepared from spleen cells and reverse transcribed into cDNA. The expression level of IFN-γ and IL-4 genes were amplified using specific primers and detected using SYBER-Green I using the real time PCR machine. Each block represents the average expression level of three mice from each group. Comparative C^T^ method (∆∆C^T^) was used to calculate the expression level of each cytokine gene relative to β-actin gene as internal control as mentioned in material and methods section.

In mice immunized with EgAgB8/2 gene, the highest RExL of IFN-γ was noticed in the group of mice immunized intramuscularly (89.81 ± 5.24). Mice immunized intranasally produced a moderate level of IFN-γ (49.05 ± 5.65) while using gene gun presented the lowest level of this cytokine (11.73 ± 0.42). Statistically significant differences in the IFN-γ RExL’s were noted when the following route pairs were compared: i.m. and i.n. (p = 0.002); i.m. and g.g. (p < 0.001), i.n. and g.g. (p = 0.003). In contrast, mice immunized with EgAgB8/2 gene using g.g. showed the highest RExL for IL-4 (49.14 ± 0.51), while the groups immunized intramuscularly and intranasally presented a low level of this cytokine (1.16 ± 0.17 and 2.50 ± 0.58, respectively). In this way, significant differences in RExL for IL-4 was observed in mice immunized using g.g. and those immunized either by the i.m. or i.n. routes (p < 0.001) but no significant difference was noted between mice immunized by the last two routes (p = 0.298). Moreover, the RExL of IFN-γ was significantly higher than IL-4 using the i.m. and i.n. route (p = 0.003 and p = 0.014, respectively) (Fig. 2).

## Discussion

The present study demonstrated clearly that different routes of immunization of mice using one EgAgB8/2 containing plasmid construct resulted in different cytokine and IgG antibody isotypes that affects Th1 or Th2 immune response.

Based on the levels of cytokine genes expression and IgG1/IgG2a ratios, a Th1 response was induced using the i.m. and i.n. routes, where IFN-γ was significantly higher than IL-4 and the IgG1/IgG2a R.I. ratio was low. In contrast, the g.g. route induced a dominant Th2 that is reflected by a high level of IL-4 compared to IFN-γ with a relatively higher IgG1/IgG2a ratio. Supportive data were reported by several studies where i.m. and g.g. routes were used for the immunization of BALB/c mice with DNA encoding for antigens derived from *Neisseria gonorrhoeae* (Zhu *et al.*, 2004), *Brucella abortus* (Velikovsky *et al.*, 2002) and *Brugia malayi* (Li *et al.*, 2004). In the latter study, immunization of BALB/c mice i.m. or by g.g. with individual four plasmids each containing one of four genes indicated that the route of DNA delivery and the nature of expressed Ag affects the direction of the immune response towards either Th1 (IgG2a predominance) or Th2 (IgG1 predominance). In contrast to the common trend, some *B. malayi* antigens elicited a Th2 response following i.m. immunization and others elicited a Th1 response following g.g. immunization.

The i.n. immunization route resulted in high IFN-γ expression indicating a Th1 bias even though the IgG2a response was not very high (Fig. 2). The present results are consistent with that reported by Dupuy *et al.* (1999) who found that splenic lymphocytes of mice i.n. immunized with a gene encoding a protein of human papilloma virus expressed high IFN-γ production indicating a Th1 response.

The explanation of this relatively common difference in T helper bias as a response for plasmid DNA immunization via various conventional routes such as the intradermal, i.m. and i.n. on one side and the g.g. bombardment on the other side is receiving much attention now. It appears that the type of response is multifactorial and affected by the route and method of DNA delivery (Scheerlinck *et al.*, 2001), dose of DNA delivered (Jones *et al.*, 2001), nature of Ag expressed from immunized gene (Dupuy *et al.*, 1999), subset of Ag presenting dendrocytes (Pulendran *et al.*, 1999) and other signals accompanying DNA delivery such as tissue injury (Liu *et al.*, 2005).

The present study shows that both i.m. and i.n. routes can evoke a stronger Th1 than Th2 response. Interest in the i.n. route as a target for DNA immunization experiments is appropriate in developing vaccines against pathogens that use the mucosal surfaces for attachment and penetration (Holmgren & Czerkinsky, 2005). This applies to infection of intermediate hosts with *E. granulosus* which occurs through oral ingestion of eggs and the penetration of the hatched oncosphere through the intestinal mucosa on its way to the blood circulation and then to filtering organs (Eckert & Deplazes, 2004). In this study, the promising results using the i.n. route indicates its potential activity in inducing a Th1 response that may protect animals as well as human infections. Further studies to elucidate direct immune response to mucosal means of antigen delivery such as IgA and cytokine expression levels in lungs of immunized mice are indicated.

Although the i.n. DNA immunization induces a Th2 response conventionally, intensive immunization with a high dose of delivered DNA appeared to switch the response toward a Th1 response (Jones *et al.*, 2001). Thus, the Th1 bias obtained in this study may be a consequence of an intense immune stimulation resulting from a probable intensive antigen expression by nasal-pulmonary cells. Further investigations to clarify this issue warrant the use of different dosages and using the same route of inoculation.

In conclusion, this study clearly demonstrated that both i.m. and i.n. route of EgAgB8 DNA immunization induce a Th1 response. These routes and their effects can be confirmed by evaluation of the protection rates generated in mice immunized against secondary hydatidosis.
